# *Marasmius oreades* agglutinin enhances resistance of Arabidopsis against plant-parasitic nematodes and a herbivorous insect

**DOI:** 10.1186/s12870-021-03186-0

**Published:** 2021-09-01

**Authors:** Aboubakr Moradi, Tina Austerlitz, Paul Dahlin, Christelle AM Robert, Corina Maurer, Katja Steinauer, Cong van Doan, Paul Anton Himmighofen, Krzysztof Wieczorek, Markus Künzler, Felix Mauch

**Affiliations:** 1 Department of Biology, University of Fribourg, Fribourg, Switzerland; 2 Institute of Plant Protection, Department of Crop Sciences, University of Natural Resources and Life Sciences, Vienna, Austria; 3 Agroscope, Research Division, Plant Protection, Phytopathology and Zoology in Fruit and Vegetable Production, Wädenswil, Switzerland; 4 Institute of Plant Sciences, University of Bern, Bern, Switzerland; 5 Oeschger Center for Climate Change Research, Bern, Switzerland; 6 Institute of Microbiology, Department of Biology, ETH Zürich, Zürich, Switzerland

**Keywords:** *Marasmius oreades* agglutinin, Arabidopsis, *Heterodera schachtii*, *Meloidogyne incognita*, *Plutella xylostella*

## Abstract

**Background:**

Plant-parasitic nematodes and herbivorous insects have a significant negative impact on global crop production. A successful approach to protect crops from these pests is the *in planta* expression of nematotoxic or entomotoxic proteins such as crystal proteins from *Bacillus thuringiensis* (*Bt*) or plant lectins. However, the efficacy of this approach is threatened by emergence of resistance in nematode and insect populations to these proteins. To solve this problem, novel nematotoxic and entomotoxic proteins are needed. During the last two decades, several cytoplasmic lectins from mushrooms with nematicidal and insecticidal activity have been characterized. In this study, we tested the potential of *Marasmius oreades* agglutinin (MOA) to furnish Arabidopsis plants with resistance towards three economically important crop pests: the two plant-parasitic nematodes *Heterodera schachtii* and *Meloidogyne incognita* and the herbivorous diamondback moth *Plutella xylostella*.

**Results:**

The expression of MOA does not affect plant growth under axenic conditions which is an essential parameter in the engineering of genetically modified crops. The transgenic Arabidopsis lines showed nearly complete resistance to *H. schachtii*, in that the number of female and male nematodes per cm root was reduced by 86–91 % and 43–93 % compared to WT, respectively. *M. incognita* proved to be less susceptible to the MOA protein in that 18–25 % and 26–35 % less galls and nematode egg masses, respectively, were observed in the transgenic lines. Larvae of the herbivorous *P. xylostella* foraging on MOA-expression lines showed a lower relative mass gain (22–38 %) and survival rate (15–24 %) than those feeding on WT plants.

**Conclusions:**

The results of our *in planta* experiments reveal a robust nematicidal and insecticidal activity of the fungal lectin MOA against important agricultural pests which may be exploited for crop protection.

**Supplementary Information:**

The online version contains supplementary material available at 10.1186/s12870-021-03186-0.

## Background

Plants encounter various biotic stresses in their environmental habitat by aboveground and belowground pests like shoot- and root-feeding herbivores and root-feeding nematodes [1]. Insect herbivores and nematodes are among the most significant threats to plant survival due to their abundance, adaptability, and diversity [2, 3]. An estimated 12.3 % (157 bio. USD) of the global crop yield is lost due to plant-parasitic nematodes [4]. Another 10–16 % of the global crop production is lost because of insect pests before harvest plus a similar degree of damage post-harvest [5].

Pest control using agrochemicals (herbicides and pesticides) has been very successful and heavily used but this approach is problematic due to the contamination of the environment with these compounds, many of which are also toxic for non-target organisms including humans [6]. An alternative approach to the use of agrochemicals in crop production is the use of genetically engineered (transgenic) crops with enhanced resistance to pests and pathogens, e.g. by *in planta* expression of heterologous entomotoxic and nematotoxic proteins [7, 8]. However, the emergence of resistances to such biopesticides reduces the efficiency of this approach. For example, field-evolved resistance of various pests has been reported in crops expressing insecticidal proteins from *Bacillus thuringiensis* (*Bt*) [9]. To solve this problem, novel entomotoxic and nematotoxic proteins which, upon *in planta* expression, furnish plants with enhanced pest resistance, are needed [8]. An attractive source for such proteins are lectins. Lectins are a widespread group of proteins binding reversibly to glycoepitopes without changing their chemical structure [10]. They are involved in a wide range of intra- and extracellular functions, including natural plant defense and immunity [11]. Accordingly, expression of heterologous plant lectins in transgenic crops is discussed and has already been applied for pest control [12, 13]. Besides plants, fruiting bodies (sporocarps) of mushrooms are a rich source of lectins with entomotoxic and nematotoxic activity. These cytoplasmically localized proteins, also referred to as fruiting body lectins, are considered as an essential part of the fungal innate defense system against predators and parasites [14, 15]. Some of these lectins were shown to recognize glycans of glycoproteins or glycolipids in the digestive tract of fungivores [14, 16]. As examples, galectins CGL1 and CGL2 from *Coprinopsis cinerea* show toxicity against the nematode *Caenorhabditis elegans*, the mosquito *Aedes aegypti*, and the amoebozoon *Acanthamoeba castellanii* [16]. Similarly, CCL2 is a β-trefoil dimeric lectin from the same mushroom that exhibits toxicity against *C. elegans*, the fruit fly *Drosophila melanogaster* and the fungivorous nematodes *Aphelenchus avenae* and *Bursaphelenchus okinawaensis* [17–19]. CCL2 also exhibits toxicity towards the animal-parasitic nematode *Haemonchus contortus* [20]. We have recently demonstrated that the expression of CCL2 in Arabidopsis protects the plants against the plant-parasitic nematode *Heterodera schachtii*. Interestingly, CCL2-expressing plants also show resistance to fungal and bacterial pathogens. Additionally, CCL2 expression promotes plant growth, suggesting that CCL2 has, besides its direct binding to glycoepitopes in the antagonist, the ability to improve plant disease resistance and biomass production via binding to endogenous glycoepitopes [21]. These results motivated us to evaluate the toxicity of another mushroom lectin, *Marasmius oreades* agglutinin (MOA), towards two different plant-parasitic nematodes and an insect herbivore. *M. oreades*, known as the fairy ring mushroom, grows in lawns, parks, pastures and meadows, and produces many bioactive compounds, such as hydrogen cyanide, polyacetylene, and several sesquiterpenes [22]. MOA is a chimerolectin containing a ricin B-type (β-trefoil) lectin domain at its N-terminus [23, 24]. The lectin domain binds specifically to Galα1,3Galβ1,4GlcNAc, which is also known as porcine xenotransplantation epitope and present in the blood group B antigen [25]. The C-terminal domain of MOA consists of a calcium-dependent cysteine protease belonging to the papain-like cysteine proteases family (PLCPs, EC3.4.22) [26]. The nematotoxicity of MOA is dependent on both the N-terminal carbohydrate-binding activity and the C-terminal cysteine protease activity, and the target in *C. elegans* has been identified as the Galα1,3Galβ1,4GlcNAc-epitope on glycosphingolipids, similar to bacterial crystal toxin Cry5B [27, 28].

This study aimed at the evaluation of the protective effect of MOA against three agronomically important plant pests. MOA-expressing Arabidopsis plants were challenged with the sugar beet cyst nematode *H. schachtii*, the root-knot nematode *M. incognita*, and the diamondback moth *Plutella xylostella*. The results demonstrate that the expression of MOA in transgenic plants can enhance their resistance towards these pests.

## Results

### Expression of MOA in Arabidopsis plants

**Fig. 1:**
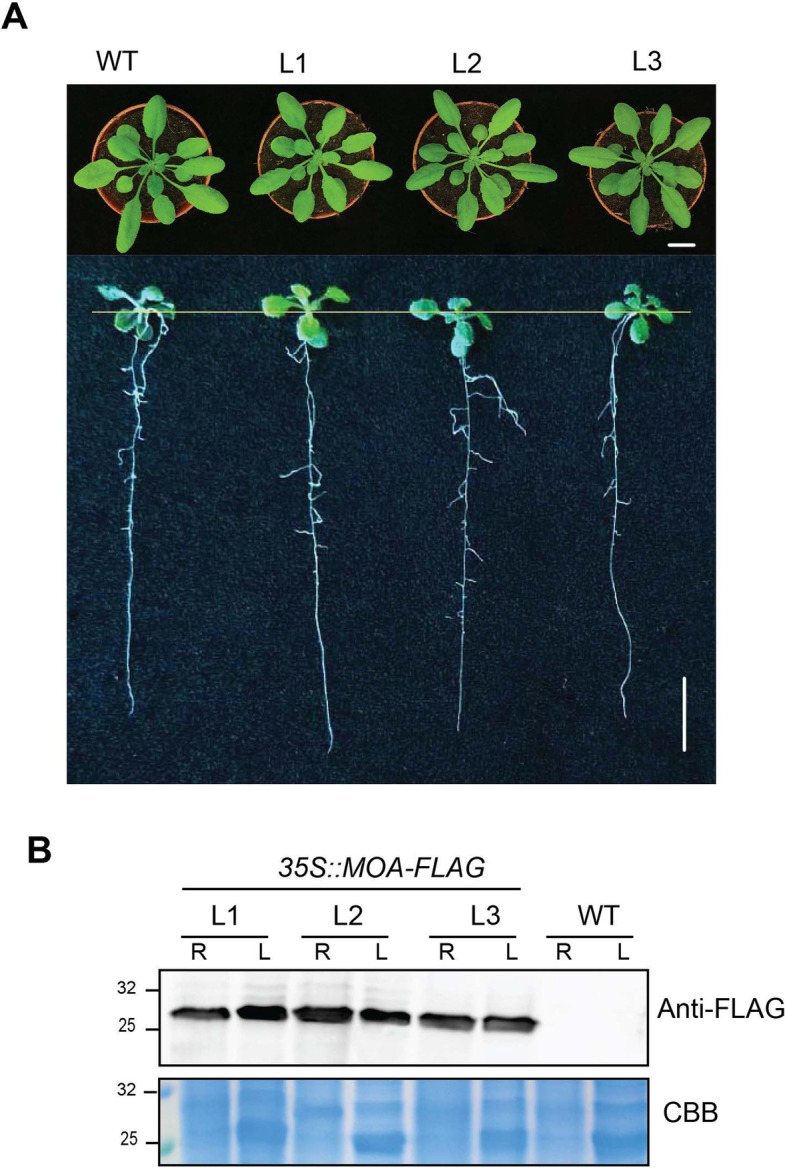
Characterization of MOA-expressing transgenic Arabidopsis lines. **a** Growth phenotype of TG lines compared to WT (Col-0) plants. The three independent lines MOA OE-lines 1, 2 and 3 (L1-L3) were selected for further experiments. The rosette and root phenotypes were assessed on four-week-old (upper panel) and 14-days-old plants (lower panel) cultivated as described in Methods. Scale bar = 1 cm. **b** Immunoblot analysis of MOA expression level in leave (L) and root (R) extracts. FLAG-tagged MOA was detected using anti-FLAG antibodies. Coomassie brilliant blue(CBB)-stained SDS-PAGE of leave and root extracts was used as a loading control. Full- length versions of the anti-FLAG- and Ponceau-S-stained blot and the CBB-stained SDS-PAGE gel are provided as additional file Fig. S1

MOA carrying a C-terminal FLAG epitope tag was expressed in Arabidopsis (accession Col-0) under the control of the constitutive CaMV-35 S promoter using the construct *35 S::MOA-3xFLAG*. Forty-two primary transformants (T1) were obtained after Arabidopsis transformation. From generation T3, three lines with high MOA expression were selected for nematode and insect infestation bioassays. The expression of MOA in Arabidopsis did not alter the size and the morphology, judged by rosette and root architecture, of the transgenic lines compared to wild-type plants (Fig. 1a). These results show that plant fitness is not affected by the expression of MOA. The level of MOA expression was analyzed in roots and leaves of three independent transgenic lines by immunoblotting (Fig. 1b). The results show that the expression level was similar in all samples analyzed.

### MOA-expressing Arabidopsis plants are more resistant to the sugar beet cyst nematode ***Heterodera schachtii***

**Fig. 2:**
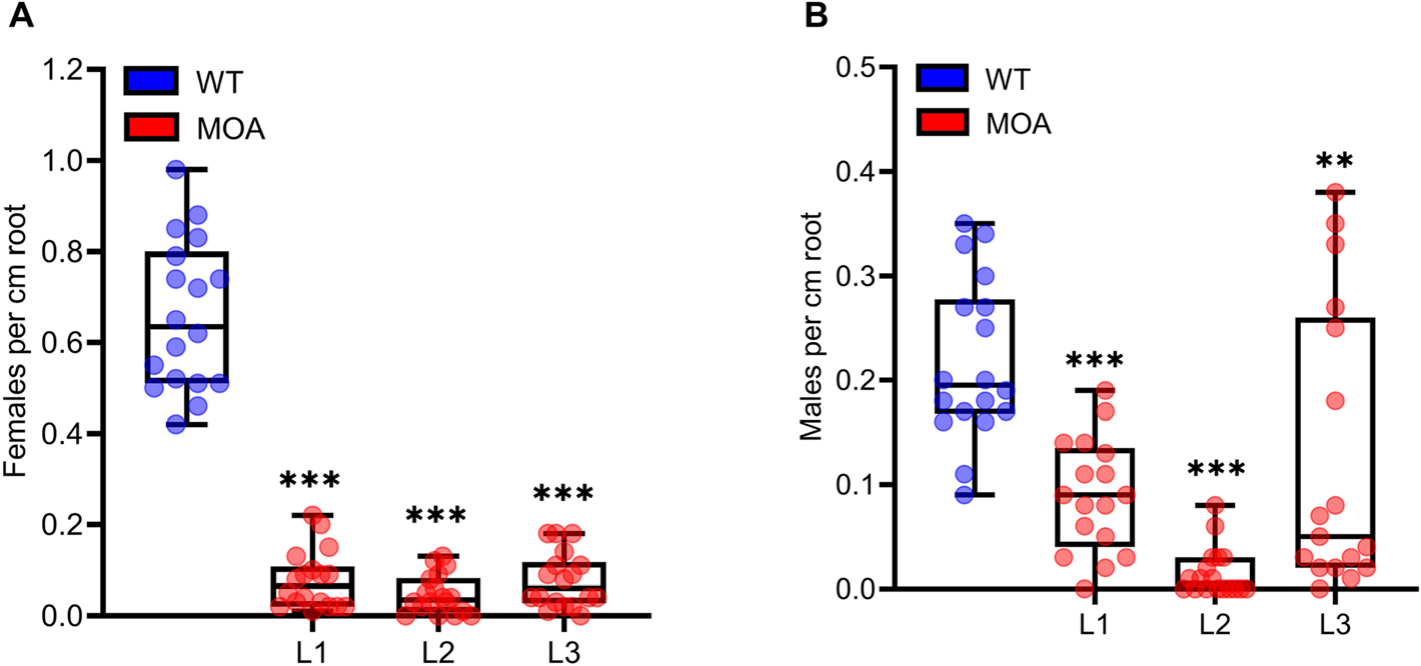
Development of the sugar beet cyst nematode *H. schachtii* on three MOA-expressing lines and WT plants. Twelve-day-old transgenic lines and WT Arabidopsis seedlings were inoculated with 30 freshly hatched juveniles (J2s) per plant. The number of female **a** and male **b** nematodes per root centimeter was evaluated at 14 dpi. Boxplots represent median and 1.5 times the interquartile range, n = 18 from three independent experiments. Asterisks above columns indicate statistically significant differences (***P ≤ 0.001, **P ≤ 0.01) between MOA-expressing lines and WT plants, analyzed by one-way ANOVA and *post-hoc* analysis with Dunnett’s multiple comparison test

The cyst nematode *H. schachtii* and the root-knot nematode *M. incognita* are sedentary endoparasitic nematodes which infect the majority of plant species including *Arabidopsis thaliana* [29, 30]. To assess the toxicity of MOA towards *H. schachtii*, MOA-expressing lines and WT plants were inoculated with second-stage juveniles (J2s) of *H. schachtii* and the number of females and males per cm of root were determined 14 days post-inoculation (dpi) to evaluate the rate of nematode infection. The number of *H. schachtii* females per cm of root was significantly reduced in all 3 MOA-expressing lines compared to WT plants (L1: 87 % protection; L2: 91 %; L3: 86 %; Fig. 2a). The number of male nematodes per cm of root also substantially decreased in MOA-expressing lines compared to WT by 55 % (L1), 93 % (L2) and 43 % (L3) (Fig. 2b). Taken together, these results demonstrate that MOA expression protects Arabidopsis plants against *H. schachtii*.

### MOA-expressing Arabidopsis plants are less susceptible to the root-knot nematode ***Meloidogyne incognita***

**Fig. 3:**
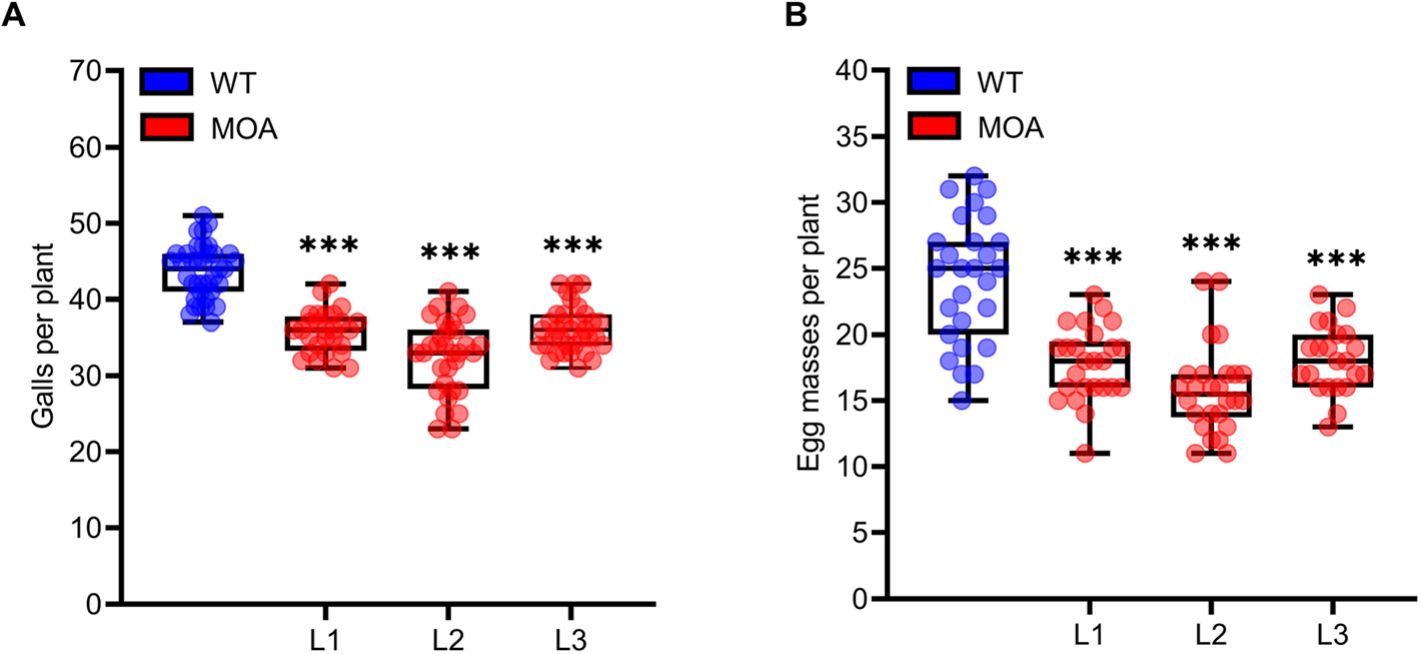
Infection and development of *M. incognita* on three MOA transgenic lines and WT plants. Three-week-old seedlings were exposed to 200 s-stage juveniles (J2s) per plant. The number of galls (**a**) and egg masses (**b**) per plant were analyzed at 35 dpi. Boxplots represent median and 1.5 times the interquartile range, (n; no. of galls, WT = 37, L1 = 28, L2 = 32, L3 = 35 and no. of egg masses, WT = 27, L1 = 25, L2 = 26, L3 = 22) from three biological replicates. Asterisks above columns indicate statistically significant differences (***P ≤ 0.001) between MOA-expressing lines and WT plants, analyzed by one-way ANOVA and *post-hoc* analysis with Dunnett’s multiple comparison test

The root-knot nematode *M. incognita* is an obligate biotrophic parasite that penetrates the plant roots and hijacks plant nutrients [31]. MOA-expressing lines and WT plants were inoculated with second-stage juveniles (J2s) of *M. incognita*. The number of galls and egg masses per plant were determined as measure of infection. The results indicate that the three transgenic lines showed a reduction in gall numbers by 18, 26 and 18 %, respectively, compared to WT plants (Fig. 3a). The number of egg masses was decreased by 26, 35 and 25 %, respectively (Fig. 3b). These results indicate that the MOA-expressing lines partially protect Arabidopsis roots from parasitism by *M. incognita.* The protective effect was, however, much weaker than with *H. schachtii*.

### MOA Enhances Arabidopsis resistance against the insect herbivore ***Plutella xylostella***

**Fig. 4:**
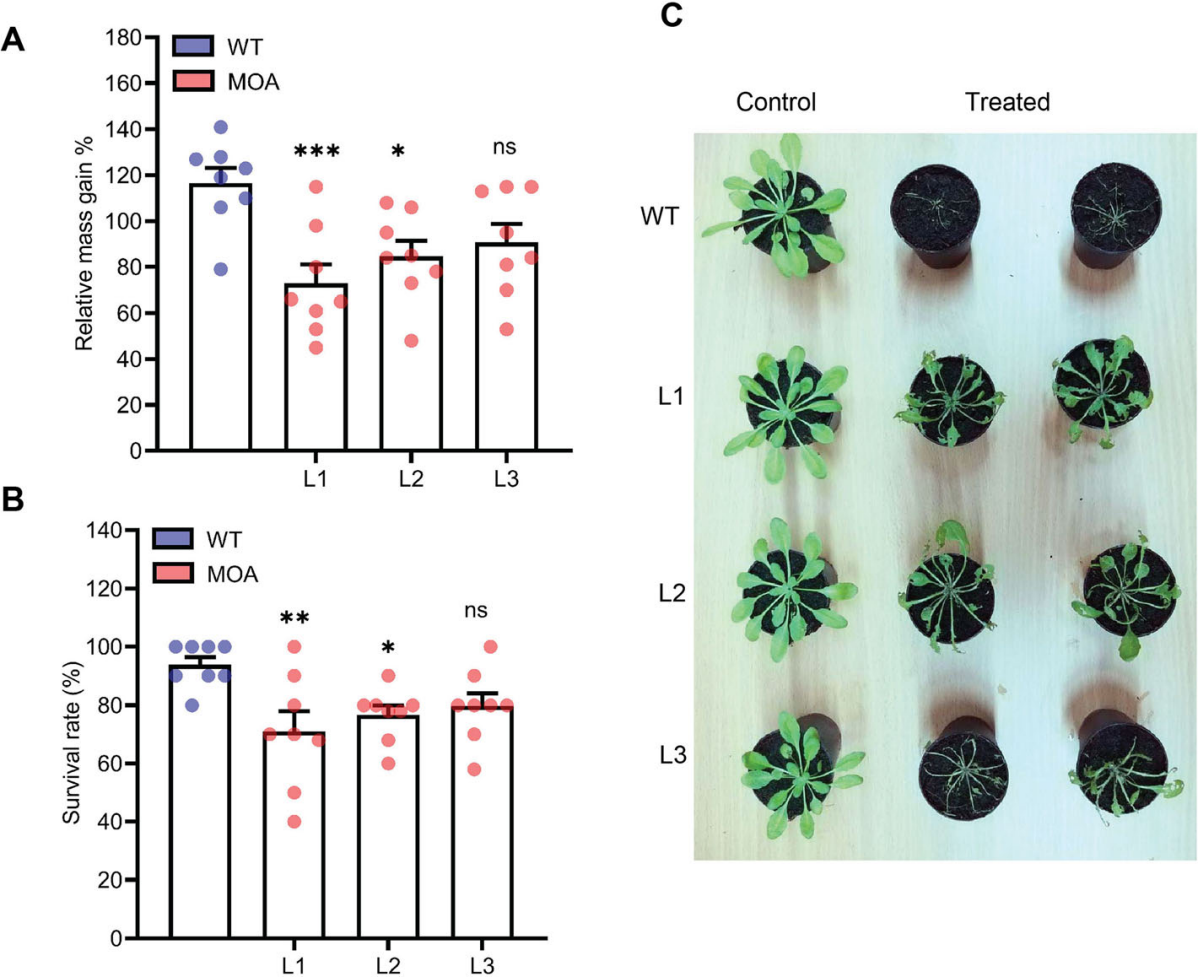
Leaf-feeding toxicity assay with larvae of *P. xylostella*. Five-week-old MOA-expressing and WT plants were exposed to larvae, 8 plants per line were inoculated with 5 pre-weighted second-instar larvae per plant. After seven days, the relative mass gain. **a** and survival rate **b** was determined. Values are means ± SE, and n = 8; mean of three biological replicates. Asterisks above columns indicate statistically significant differences compared to control (***P ≤ 0.001, **P ≤ 0.01, *P ≤ 0.05, ns: not significant) between MOA-expressing lines and WT plants, analyzed by one-way ANOVA and *post-hoc* analysis with Dunnett’s multiple-comparison test. **c** Phenotype of plants treated with larvae. Control: Plants were incubated under the same conditions without larvae. Treated: Plants were inoculated with larvae as described above. Pictures were taken 7 days post-inoculation

Herbivorous insects, such as the diamondback moth *Plutella xylostella*, contribute to an estimated loss of 15 % of global crop production [32]. Five-week-old MOA-expressing plants and WT plants were exposed to *P. xylostella* larvae for a week and the larval relative weight gain and survival were analyzed. The results indicate that the relative mass gain in all MOA-expressing lines was lower compared to WT plants. Larval mass gain was reduced by 38 % in L1, 27 % in L2 and 22 % in L3 (Fig. 4a). Similarly, larvae feeding on L1 to L3 have 24 %, 18 and 15 % lower survival rates compared to WT plants (Fig. 4b). MOA-OE L1, with the highest MOA expression in the leaves, showed better inhibition against the leaf-eating insect compared to L2 and L3. The phenotype of the treated plants shows that WT plants were entirely eaten by larvae, whereas the transgenic lines showed some resistance to larval infestation (Fig. 4c). The results demonstrate the potential of MOA for controlling infestation by *P. xylostella*.

## Discussion

In this study, we tested the mushroom lectin MOA [24] for its capability to enhance the resistance of engineered transgenic Arabidopsis plants towards two different plant-parasitic root nematodes and a herbivorous shoot insect herbivore. Expression of resistance genes can negatively affect plant growth. For example, the expression of glyphosate resistance genes in plants causes fitness cost [33]. Our results show that the expression of MOA does not reduce the fitness of Arabidopsis plants under axenic conditions (Fig. 1a). In this regard, it might be an advantage that mushroom lectins, in contrast to many plant lectins, are produced in the cytoplasm, a compartment that is very poor in endogenous glycoconjugates.

Plant-parasitic nematodes have a substantial negative impact on agriculture causing a loss of over US$ 100 billion annually [3]. As these parasites spend part of their life cycle inside the plant tissue, control by applying chemical pesticides is challenging. Here, the application of transgenic plants expressing the biopesticides in the plant tissue is a valuable and much more effective alternative. Our results show that MOA-expressing Arabidopsis plants were substantially protected against the cyst nematode *H. schachtii* (Fig. 2). All three lines displayed a significant reduction in the number of female and male nematodes per cm of root. We have previously demonstrated that the CCL2-expressing Arabidopsis plants are resistant to *H. schachtii* in that the number of females per cm of root was reduced by 35 % in CCL2-expressing lines compared to WT plants. The protective effect of MOA (reduction by 88 %) is 60 % higher than the one of CCL2.

Our MOA transgenic lines showed also reduced susceptibility to the root-knot nematode *M. incognita* albeit to a much lower extent than to *H. schachtii* (Fig. 3). This difference might be due to a lower susceptibility of *M. incognita* to MOA or due to a lower exposure of the nematode to the lectin due to a differences in the life cycle. Our results are in line with the results of Ripoll et al. [34], who expressed GNA, a mannose-binding lectin from snowdrop plant (*Galanthus nivalis*), in Arabidopsis and exposed plants to *M. incognita* J2s. Only three out of nine lines showed a significant decrease in gall number (20–50 %) compared to the WT plants. Moreover, expression of another fungal lectin (SRL) has recently been shown to provide protection to tomato plants towards *M. incognita* [35]. The observed reductions in number of galls and female nematodes in the transgenic tomato lines were similar to our results.

The damage of the leaf chewing insect *P. xylostella*, one of the most devastating pests of cruciferous vegetables, was estimated at more than US$ 5 billion annually [36, 37]. Thus, controlling these pests needs special attention to maintain and improve food production. The results demonstrate that *in planta* expression of MOA has a protective effect against *P. xylostella* larvae. The larvae were vulnerable to MOA, in that they gained less weight and had a higher mortality rate compared to the WT-feeding larvae. So far, transgenic crops against diamondback moth are based on the *B. thuringiensis cryIA(b)* gene [38] but mammalian and viral lectins have been shown to exert some toxicity towards this pest [39, 40]. Our study is, to our knowledge, the first evidence for the toxicity of a fungal lectin against *P. xylostella*. These results are in line with reports about other fungal lectins, e.g. *Rhizoctonia solani* agglutinin (RSA; β-trefoil-type lectin) and *S. rolfsii* lectin (SRL; actinoporin-type lectin), which show substantial toxicity against agriculturally important herbivorous insects such as cotton leafworms *Spodoptera littoralis* and *S. litura* [41, 42]. Interestingly, SRL-expressing plants showed also resistance to sucking and chewing insects *S. litura* and *Myzus persicae* [43]. Accordingly, SRL-transgenic cotton plants showed high resistance to *Aphis gossypii* (69 % reduction in population) and *S. litura* (100 % larval mortality) [44]. These studies suggest that MOA may provide protection against other insect herbivores.

Further experiments will be needed to clarify the mechanism of action and the specificity of MOA. The toxicity towards *C. elegans* relies on both the lectin and the protease domain of the chimerolectin [27]. It will be interesting to find out whether the mechanism of action is the same for the plant-parasitic nematodes and the herbivorous insect. In this regard, a serine protease, Sep1, from *Bacillus firmus* DS-1 has recently been shown to possess nematicidal activity against *M. incognita*. The purified protein caused 50.36 % mortality on J2 animals at 500 µg/ml [45]. For determining the specificity, MOA-transgenic plants will be exposed to a wider range of insect herbivores, such as leaf-chewing and sap-sucking, insects.

## Conclusions

Taken together, our results demonstrate that MOA-expressing Arabidopsis plants show increased resistance to several economically important pests: the devastating plant-parasitic nematodes *H. schachtii* and *M. incognita* as well as the herbivorous insect *P. xylostella*. We conclude that the mushroom lectin MOA has a high potential for being applied for the engineering of insect/nematode-resistant transgenic crops.

## Methods

### Plant growth conditions

Wild type Arabidopsis accession Columbia-0 (Col-0) were obtained from the Nottingham Arabidopsis Stock Centre (U.K.). Wild type and transgenic plants were sown into Jiffy artificial soil (Jiffy International AS, Norway). After stratification at 4 °C for three days, the plants were transferred to growth chambers under the following conditions: 22.5‎°C/19°C day/night temperature under 16 h of light (photon flux density of 100 µmmol m^− 2^ s^− 1^) with 60 % relative humidity. To evaluate the root architecture of the transgenic plants, the seeds were surface-sterilized [46] and grown on ½ Murashige and Skoog (MS) medium containing 3 % sucrose under above conditions. The seedlings were photographed 14 days after their transfer to MS medium.

### Construction of plant expression vectors

A construct expressing C-terminally FLAG-tagged MOA under the control of the CaMV 35 S promoter was generated using the Gateway R Cloning Technology (Thermo Fisher Scientific, USA). The open reading frame of MOA was PCR-amplified using gene-specific primers (MOA-Fw: 5′-CACCATGTCTCTGCGACGCGG-3′, and MOA-Rev: 5′-GTAGAAGGCCATGTAGCTGTC-3′) from pET-22b(+) plasmid (Merck Millipore Novagen, USA). The PCR product was introduced into a pENTR vector (pENTR™/D-TOPO™ Cloning Kit, Thermo Fisher Scientific, USA) and the reaction products were transformed into chemically competent TOP10 *E. coli* cells. Positive colonies were detected by colony PCR (Biometra, Germany). The DNA sequence was confirmed by sequencing (Eurofins Genomics, Germany). The obtained entry plasmids were recombined into the binary Gateway expression vector pB2GW7 [47], utilizing LR reaction (Gateway™ LR Clonase™ II Enzyme mix, Thermo Fisher Scientific, USA). The colony PCR-confirmed binary expression plasmid (*35 S::MOA-3xFLAG*) was transformed into *Agrobacterium tumefaciens* strain GV3101 by the freeze-thaw method [48].

### Expression of MOA in Arabidopsis plants

*Agrobacterium tumefaciens*–mediated transformation of WT plants utilizing the floral dip method was performed as described [49]. Transformed plants were identified on MS medium containing 15 µg mL^− 1^ of glufosinate-ammonium (Basta®, Bayer CropScience AG, Germany). Healthy seedlings were transferred to soil to determine the protein expression levels by standard immunoblotting methods. Briefly, thirty mg tissue of four-week-old plants were collected and frozen in liquid nitrogen. The frozen tissue in 1.5-mL Eppendorf® tubes (Eppendorf, Germany) containing two 3-mm glass beads was ground with a mixer mill (Retsch® MM400, Retsch Technology GmbH, Germany) adjusted at 30 Hz for 3 min. Subsequently, 90 µL of preheated Laemmli buffer (375 mM Tris-HCl, pH 6.8, 37 % glycerol, 0.06 % bromophenol blue sodium salt, 12 % sodium dodecyl sulfate, and 5 % β-mercaptoethanol) was added to the tubes. The samples were incubated for 10 min at 95 °C with agitation (1400 rpm) and centrifuged at maximum speed (14,000 rpm) for 10 min. Protein concentration was estimated by the Pierce™ BCA Protein Assay Kit (Thermo Fisher Scientific, USA). Ten µL of the supernatant (2 µg mL^− 1^ of crude extract) was used for SDS-PAGE. The separated proteins were transferred to nitrocellulose membranes (Merck KGaA, Darmstadt, Germany) with a Mini Trans-Blot® Cell (Bio-Rad Laboratories, California, USA). For immunoblotting, membranes were blocked with 3 % milk in TBST buffer (150 mM NaCl, 10 mM Tris, 0.1 % (v/v) Triton X-100, pH 7.6). Anti-FLAG primary antibodies (1:1000; monoclonal anti-FLAG M2-Peroxidase (HRP) clone M2, (Merck KGaA, Germany) were used to detect FLAG-tagged proteins. Pierce™ ECL Western Blotting Substrate (Thermo Fisher Scientific, USA) and horseradish peroxidase (HRP) were used for blot development. Signals were detected by ImageQuant Las 4000 (GE Healthcare Life Sciences, USA). Transgenic seeds were surface-sterilized [46]. The seeds were grown on ½ Murashige and Skoog medium (MS) with an appropriate selection marker and incubated in the described condition. Three-week-old seedlings were removed from the media, and 30 mg of fresh tissue was used to determine expression level in leaves and roots of each line as described above. From 42 transgenic plants, three independent lines were chosen for further experiments. Expression analysis and all disease resistance tests were performed with glufosinate-ammonium selected T3 generation plants.

### ***Heterodera schachtii*** stock culture and infection assay

Arabidopsis seeds (Col-0 and MOA L1-3) were surface-sterilized for 1 min in 70 % (vol/vol) ethanol, submerged for 8 min in 2.8 % (w/v) sodium hypochlorite, subsequently washed three times for 3 min in sterile distilled H_2_O and dried overnight. The lines were grown on selective MS medium supplemented with 3 % sucrose and 10 mg L^− 1^ glufosinate-ammonium. WT plants were grown on MS medium lacking glufosinate-ammonium. After five days, healthy seedlings were moved to plates containing a modified 0.2 concentrated Knop medium supplemented with 2 % sucrose [29] and transferred to a growth chamber at 24 °C with an 18 h of light for another seven days (12 days in total). Six plates per line with eight plants per plate were prepared. The experiment was repeated three times.

*H. schachtii* infection assay was carried out as described previously [50]. Cysts of *H. schachtii* were harvested from *in vitro* stock cultures on mustard roots (Sinapsis alba ‘Albatros’) growing on 0.2 concentrated Knop medium supplemented with 2 % sucrose [29]. Hatching of 2nd stage juveniles (J2s) was stimulated by soaking cysts in 3 mM ZnCl_2_. The juveniles were additionally surface-sterilized with 0.05 % HgCl_2_ for 2 min, washed three times in sterile H_2_O and resuspended in 0.7 % (w/v) Gelrite (Duchefa, the Netherlands). Before inoculation, the total root length of each line was estimated according to described protocol [51]. For infection assays, 12-day-old plants were infected with 30 freshly hatched juveniles per plant, subsequently kept in the dark overnight, transferred to the growth chamber at 21 °C with a 12 h/12 h day/night cycle. The nematode infection was evaluated 14 days post nematode inoculation (dpi). The total number of females and males was counted and the number of females and males per root cm was calculated.

### ***Meloidogyne incognita*** culture and infection assay

*M. incognita* was reared on tomato (*Solanum lysopericum* cv. Oskar) plants [52] growing under greenhouse conditions, with a day/night cycle of 15:9 h; 24 ± 2 °C and 60 % relative humidity. J2s were stimulated to hatch and extracted under the mist chamber, after removal of the sandy soil from the tomato roots. Hatched J2s were collected over one week and stored at 4 °C. A 40X magnification was used on an inverted light microscope for J2 quantification and preparation of the nematode suspension.

Arabidopsis seeds (Col-0 and MOA L1-3) were pre-germinated on MS medium containing 15 µg mL^− 1^ glufosinate-ammonium. The healthy seedlings were transplanted to pots containing silver sand:soil (4:1; v/v) and grown at 22 °C, 60 % relative humidity with a day-night cycle of 16:8 h. The plants were watered with autoclaved tap water, containing 1:1000 H_2_O: fertilizer dilution (v/v; Wuxal, Hauert, Switzerland). Three-week-old plants were exposed to 200 J2s of *M. incognita*. At 35 dpi roots were washed carefully to remove substrate, incubated in 1 % food coloring Ponceau 4R (E 124) for 10 min and distained in tap water for 15 min. The egg masses were counted under a stereo binocular dissecting microscope at 30X magnification [53]. The same roots were evaluated for gall formation. The experiments were repeated two times with similar results.

### Insect herbivore toxicity assay

The effects of MOA on herbivory were tested on the leaf herbivore, *Plutella xylostella*. The performance and survival rate of larvae when feeding on the transgenic and WT plants were evaluated. *P. xylostella* eggs were kindly provided by Syngenta (Syngenta Crop Protection AG, Switzerland). The eggs were reared on artificial diet containing; 16 % Beet Armyworm Diet, 1 % USDA Vitamin Premix, Chlortetracycline 200 µg mL^− 1^ (Frontier Agriculture science, USA) and 1 % Agar (Merck KGaA, Germany) in chambers at 24 °C, 60 % relative humidity, with a 16 h light ⁄ 8 h dark photoperiod. For the insect-feeding assay, transgenic and WT plants were grown in soil (Klasmann-Deilmann GmbH, 49, Germany) in individual pots (pots Ø 6 cm, 5.5 cm height; Pöppelmann, Germany) under 22.5‎°C day/19°C night temperature and 18 h of light photoperiod (photon flux density100 µmmol m^− 2^ s^− 1^) with 60 % relative humidity. The five-week-old plants (n = 8 plants per line) were exposed to five pre-weighed second-instar larvae for a week. Then, the larvae were collected and weighed to determine their survival rate and individual relative weight gain. The experiment was repeated three times.

### Statistical analysis

Statistical analyses were carried out using Microsoft Excel and GraphPad Prism version 8.0.2 (GraphPad Software, Inc., USA). One-way ANOVA analysis was performed to identify significant differences between treatments relative to the control. Dunnett’s test was used for multiple comparisons between the lines and treatments. Asterisks indicate statistically significant differences (***P ≤ 0.001, **P ≤ 0.01, *P ≤ 0.05) whereas ns (not significant) indicates *P* > 0.05.

## Supplementary Information

**Additional file 1:Figure S1.** Immunoblot analysis visualizing the expression level of MOA in leave (L) and root (R) crude extracts. The blot was exposed in different timepoints: 1s (A), 10s (B) and 20s (C). FLAG-tagged proteins were detected with anti-FLAG antibodies. (D) Ponceau-S stained immunoblot was used as a blotting control. (E) Coomassie brilliant blue stained SDS-PAGE was used as a loading control. Molecular weights of marker proteins (M) are indicated.

## Data Availability

The raw data of the presented results of this study are available on request to the corresponding author.

## Abbreviations

Bt: *Bacillus thuringiensis*
MOA: *Marasmius oreades* agglutinin
CGL1: *Coprinopsis cinerea* Galectin1
CGL2: *C. cinerea* Galectin2
CCL2: *C. cinerea* lectin2
PLCPs: Papain-like cysteine proteases
Cry: Bacterial crystal toxin
OE: Overexpression
J2: Second-stage juvenile
L: Line
WT: Wild type
GNA: *Galanthus nivalis* lectin
RVL: *Remusatia vivipara* lectin
SRL: *S. rolfsii* lectin
RSA: *Rhizoctonia solani* agglutinin
