# Supplementary material for: Marasmius oreades agglutinin enhances resistance of Arabidopsis against plant-parasitic nematodes and a herbivorous insect
Source: BMC Plant Biol. 2021 Sep 1;21:402. doi: 10.1186/s12870-021-03186-0 (PMC8408931; doi:10.1186/s12870-021-03186-0)
Supplement: Supplementary file 1 — Additional file 1:Figure S1. Immunoblot analysis visualizing the expression level of MOA in leave (L) and root (R) crude extracts. The blot was exposed in different timepoints: 1s (A), 10s (B) and 20s (C). FLAG-tagged proteins were detected with anti-FLAG antibodies. (D) Ponceau-S stained immunoblot was used as a blotting control. (E) Coomassie brilliant blue stained SDS-PAGE was used as a loading control. Molecular weights of marker proteins (M) are indicated. [file 12870_2021_3186_MOESM1_ESM.pdf]

# SUPPLEMENTARY MATERIAL

# *Marasmius oreades* Agglutinin Enhances Resistance of Arabidopsis Against Plant-Parasitic Nematodes and a Herbivorous Insect


Aboubakr Moradi[1*], Tina Austerlitz[2], Paul Dahlin[3], Christelle AM Robert[4,5], Corina Maurer[4], Katja Steinauer[4], Cong van Doan[4], Paul Anton Himmighofen[4], Krzysztof Wieczorek[2], Markus Künzler[6*] and Felix Mauch[1]

[1]Department of Biology, University of Fribourg, Fribourg, Switzerland

[2]Institute of Plant Protection, Department of Crop Sciences, University of Natural Resources and Life Sciences, Vienna, Austria

[3]Agroscope, Research Division, Plant Protection, Phytopathology and Zoology in Fruit and Vegetable Production, 8820 Wädenswil, Switzerland

[4]Institute of Plant Sciences, University of Bern, Bern, Switzerland

[5]Oeschger Center for Climate Change Research, Bern, Switzerland

[6]Institute of Microbiology, Department of Biology, ETH Zürich, Zürich, Switzerland

***Correspondance:**

Email: aboubakr.moradi@unifr.ch Tel: +41 26 300 8831

Email: mkuenzle@ethz.ch Tel: +41 44 632 4925

**A** **Signals** **Background**

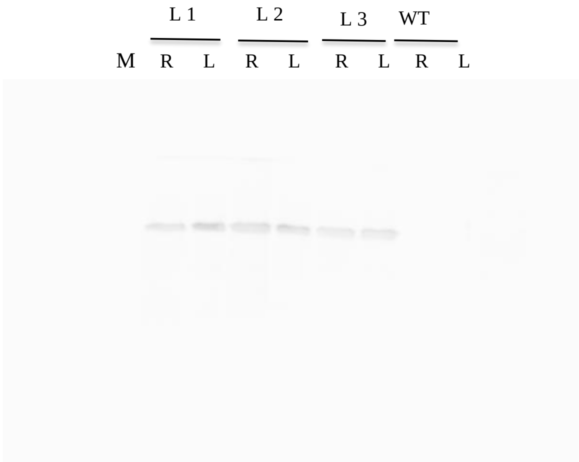


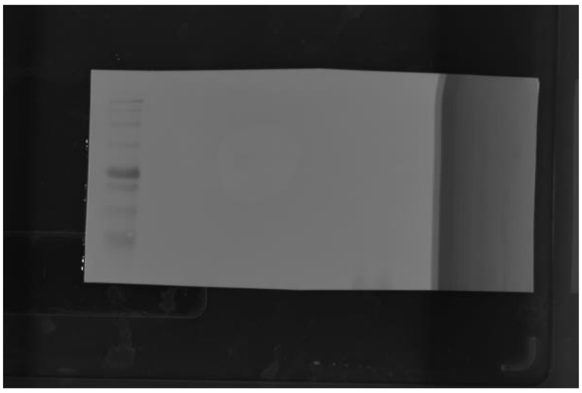

**B** **Signals** **Background**

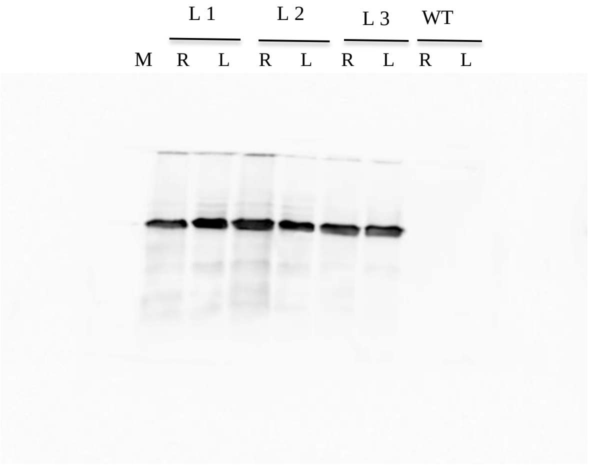


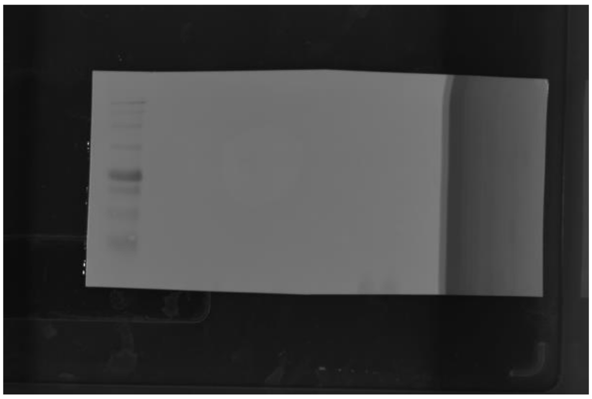

**C** **Signals** **Background**

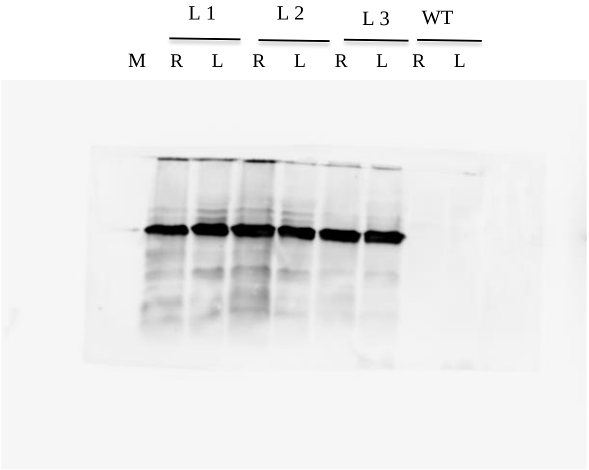


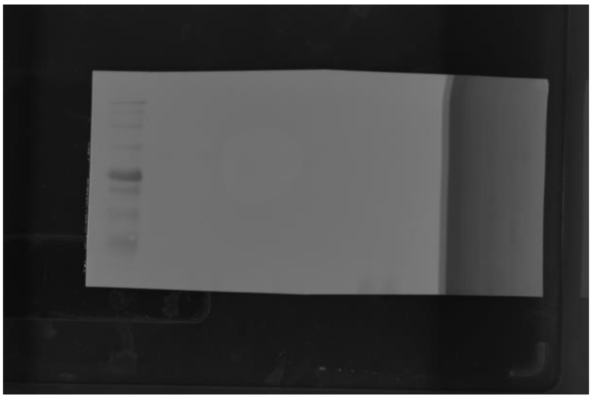

D

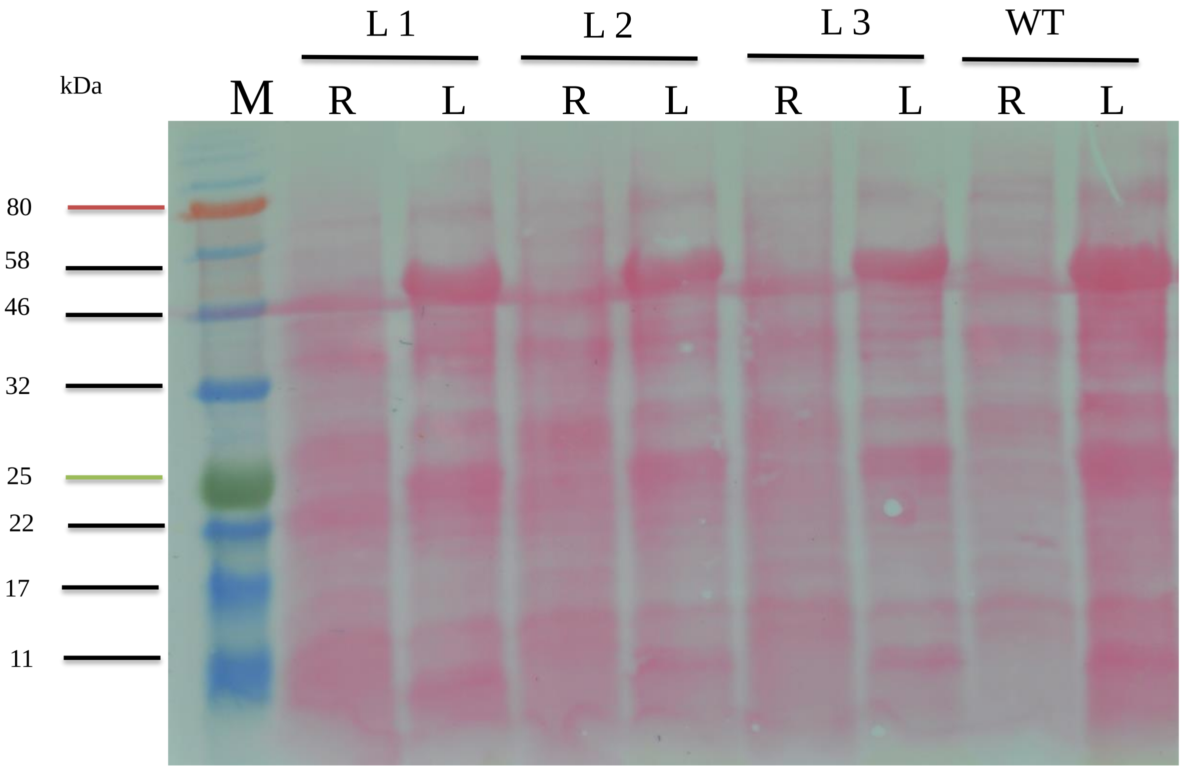
L 1
L 2
L 3
WT
kDa
M
R
L
R
L
R
L
R
L
80
58
46
32
25
22
17
11

E

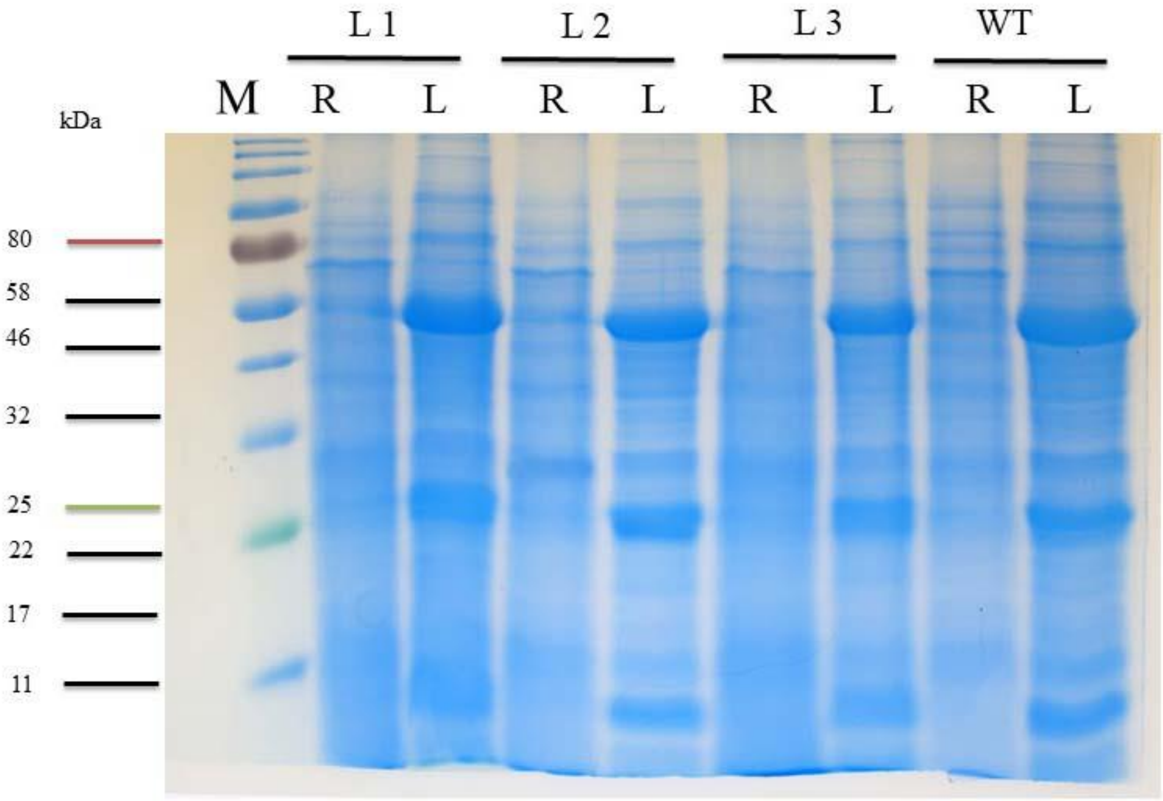
L 1
L 2
L 3
WT
M
R
L
R
L
R
L
R
L
kDa
80
58
46
32
25
22
17
11

**Figure S1.** Immunoblot analysis visualizing the expression level of MOA in leave (L) and root (R) crude extracts. The blot was exposed in different timepoints: 1s (A), 10s (B) and 20s (C). FLAG-tagged proteins were detected with anti-FLAG antibodies. (D) Ponceau-S stained immunoblot was used as a blotting control. (E) Coomassie brilliant blue stained SDS-PAGE was used as a loading control. Molecular weights of marker proteins (M) are indicated.
